# A New Electromagnetic Navigation System for Pedicle Screws Placement: A Human Cadaver Study at the Lumbar Spine

**DOI:** 10.1371/journal.pone.0133708

**Published:** 2015-07-29

**Authors:** Patrick Hahn, Semih Oezdemir, Martin Komp, Athanasios Giannakopoulos, Roderich Heikenfeld, Richard Kasch, Harry Merk, Georgios Godolias, Sebastian Ruetten

**Affiliations:** 1 Center for Spine Surgery and Pain Therapy, Center for Orthopaedics and Traumatology of the St. Elisabeth Group – Catholic Hospitals Rhein-Ruhr, St. Anna Hospital Herne/Marienhospital Herne University Hospital/Marien Hospital Witten, Herne, Germany; 2 Clinic for Orthopaedics and Orthopaedic Surgery, University Medicine of Greifswald, Greifswald, Germany; 3 Center for Orthopaedics and Traumatology of the St. Elisabeth Group – Catholic Hospitals Rhein-Ruhr, St. Anna Hospital Herne/Marienhospital Herne University Hospital/Marien Hospital Witten, Herne, Germany; University of Michigan, UNITED STATES

## Abstract

**Introduction:**

Technical developments for improving the safety and accuracy of pedicle screw placement play an increasingly important role in spine surgery. In addition to the standard techniques of free-hand placement and fluoroscopic navigation, the rate of complications is reduced by 3D fluoroscopy, cone-beam CT, intraoperative CT/MRI, and various other navigation techniques. Another important aspect that should be emphasized is the reduction of intraoperative radiation exposure for personnel and patient.

The aim of this study was to investigate the accuracy of a new navigation system for the spine based on an electromagnetic field.

**Material and Method:**

Twenty pedicle screws were placed in the lumbar spine of human cadavers using EMF navigation. Navigation was based on data from a preoperative thin-slice CT scan. The cadavers were positioned on a special field generator and the system was matched using a patient tracker on the spinous process. Navigation was conducted using especially developed instruments that can be tracked in the electromagnetic field. Another thin-slice CT scan was made postoperatively to assess the result. The evaluation included the position of the screws in the direction of trajectory and any injury to the surrounding cortical bone. The results were classified in 5 groups: grade 1: ideal screw position in the center of the pedicle with no cortical bone injury; grade 2: acceptable screw position, cortical bone injury with cortical penetration ≤ 2 mm; grade 3: cortical bone injury with cortical penetration 2,1-4 mm, grad 4: cortical bone injury with cortical penetration 4,1-6 mm, grade 5: cortical bone injury with cortical penetration >6 mm.

**Results:**

The initial evaluation of the system showed good accuracy for the lumbar spine (65% grade 1, 20% grade 2, 15% grade 3, 0% grade 4, 0% grade 5). A comparison of the initial results with other navigation techniques in literature (CT navigation, 2D fluoroscopic navigation) shows that the accuracy of this system is comparable.

**Conclusion:**

EMF navigation offers a high accuracy in Pedicle screw placement with additional advantages compared to other techniques. The short set-up time and easy handling of EMF navigation should be emphasized. Additional advantages are the absence of intraoperative radiation exposure for the operator and surgical team in the current set-up and the operator’s free mobility without interfering with navigation. Further studies with navigation at higher levels of the spine, larger numbers of cases and studies with control group are planned.

## Introduction

Pedicle screw instrumentation is one of the standard techniques in spine surgery. The correct position of the screws ensures good pullout strength in the bone and good control of rotation and possibility of repositioning the respective vertebral body [1]. The technique was first reported by Harrington [2]. The precondition for this is the correct position of the pedicle screws [3]. Faulty positioning can lead to injury to neural structures and to injuries in the chest and abdominal region. Typical clinical manifestations of this are radiculopathies, neuropathies, and epidural hematomas. A faulty position also poses an increased risk of instability [4–6]. Utmost accuracy in positioning the screws is therefore indispensable. In literature, the rate of faulty screw positions is reported to be 10% to 40% and the rate of required revisions is up 6.6% [7, 8]. The precondition for the correct placement of the pedicle screw, in addition to the correct selection of the entry point in the pedicle and the correct direction of trajectory, is the assessment of the anatomical variability of the various segments of the spine [9, 10]. Preoperative tomographic imaging is therefore recommended.

To achieve an optimal central position of the pedicle screw in the pedicle with the standard technique without injuring the medial, lateral, cranial, or caudal wall of the pedicle, it is necessary to select the correct point of insertion of the pedicle screw into the vertebra, correct spatial placement of the screw through the pedicle, and perfect fit of the diameter of the screw to the diameter of the pedicle. The optimal screw diameter can be estimated individually based on the pre-operative CT or MRI images. The correct point of insertion of the screw is determined intra-operatively as in the conventional technique using anatomical landmarks. Two different approaches to the pedicle are available: the Roy-Camille or the Weinstein approach. Precise positioning of the screws in the pedicle is essential for the stability of the pedicle systems.

From a biomechanical standpoint, the pedicle screws should ideally be placed along the pedicle axis. This makes it possible to utilize the greatest transversal and sagittal pedicle diameter and insert thicker pedicle screws. The stability of the screw in the bone is determined to a large extent by the thickness of the screw and its length inside the vertebra. Stability is increased by a greater screw diameter and insertion depth. In addition to the biomechanical aspect of stability, the ideal position also ensures optimal protection of adjacent structures

Technical advances have made several different techniques for placing pedicle screws available [8]. In addition to free-hand placement based on anatomical landmarks and intraoperative, fluoroscopic-guided placement, surgeons today also have 3D imaging methods (O-arm, 3D fluoroscopy) and various navigation systems available to them. The goal of all technical methods and advances is to increase the accuracy of screw placement [9–13]. The goal for the future is also to combine the navigation techniques with other minimal invasive procedures [11, 12]. The disadvantage of the intraoperative fluoroscopic free-hand technique is the higher rate of faulty placements and the increased radiation dose to patient and surgeon [13–15]. Intraoperative tomographic imaging results in a higher radiation dose for the patient [16].

Commercially available navigation systems can be classified into active and passive systems, i.e. they either transmit or receive signals [17, 18]. Among the preoperative 3D-based techniques, there are those known as optoelectronic methods [19] and electromagnetic navigation methods [20–22]. The disadvantages of the optoelectronic techniques are the dynamic reference basis (DRB) required for tracking and the active (LEDs) and passive (reflector balls) signal emitters attached to it.

Electromagnetic navigation uses electromagnetic fields that penetrate the body [20, 22, 23]. This allows the problem of interrupting the line of sight in conventional optical measurement systems to be avoided. Furthermore, the reference bodies are ergonomically superior to optical bodies. The aim of this experimental human cadaver study was to investigate the accuracy of pedicle screw placement in the human lumbar spine using a new EMF navigation system.

## Material and Method

### Ethics Statement

This study was conducted in compliance with the strict ethical guidelines for human cadaver studies. All body donors were fully legally competent and had a will in which they agreed to the use of their body or body parts for research, study, or teaching purposes (Section for Clinical Anatomy, Heinrich Heine University, Düsseldorf, Germany). The ethics committee of the medical Association of Westfalen-Lippe gave its approval for this study (214-037-f-S).

### Bodies Used

The study was conducted on two human bodies (1x male, 1x female) with intact spines that were preserved using the Thiel method (Section for Clinical Anatomy, Heinrich Heine University, Düsseldorf, Germany). The bodies were examined in advance by a CT scan to check for previous operations on the spine, pathological changes, tumors, and severe anomalies.

### EMF Navigation System

A new EMF navigation system with clinical approval for ENT and neurosurgery (fiagon GmbH, Hennigsdorf, Germany) and adapted for spine surgery was tested. Special instruments were developed (pedicle opener, bone awl, pedicle sounder) to make navigation in the EM field possible.

The principle of the new navigation system based on an electromagnetic measuring system is the continuous tracking of the instruments and the patient’s anatomical structures during the surgical procedure. The navigation system thus supports the operative procedure by providing additional information without changing or hindering the operator’s conventional workflow. The calibration and correction process can correct static errors that occur when measuring the position. The method takes errors into account that are attributable to the position and direction of the navigated instrument. This improves the accuracy of the position in a space adapted to the application. Navigation takes place virtually in real time in a 3D data set. Theoretically, precise intraoperative evaluation is thus possible in all planes.

### Tracking System and Instruments

**Fig 1:**
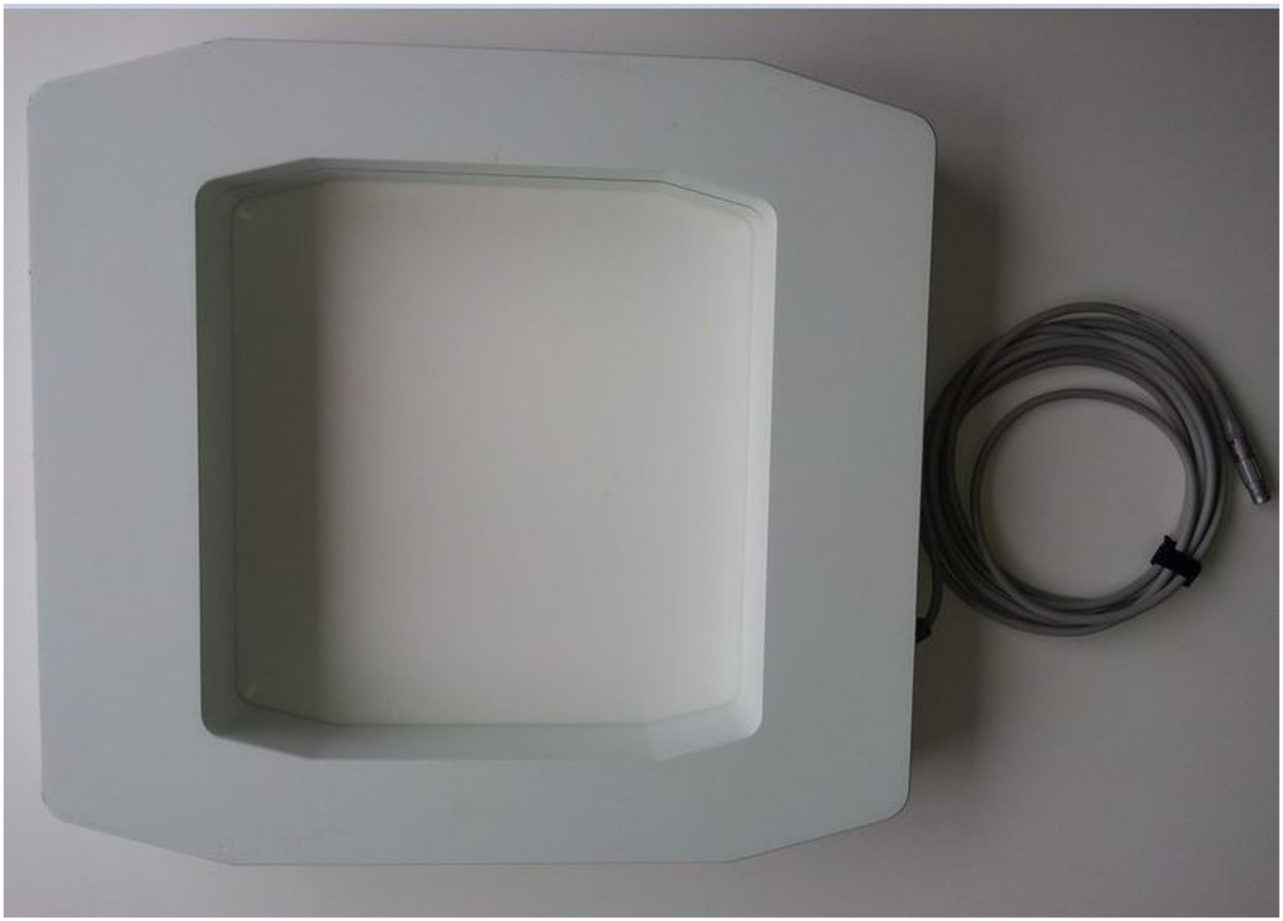
EM-Field Generator.

**Fig 2:**
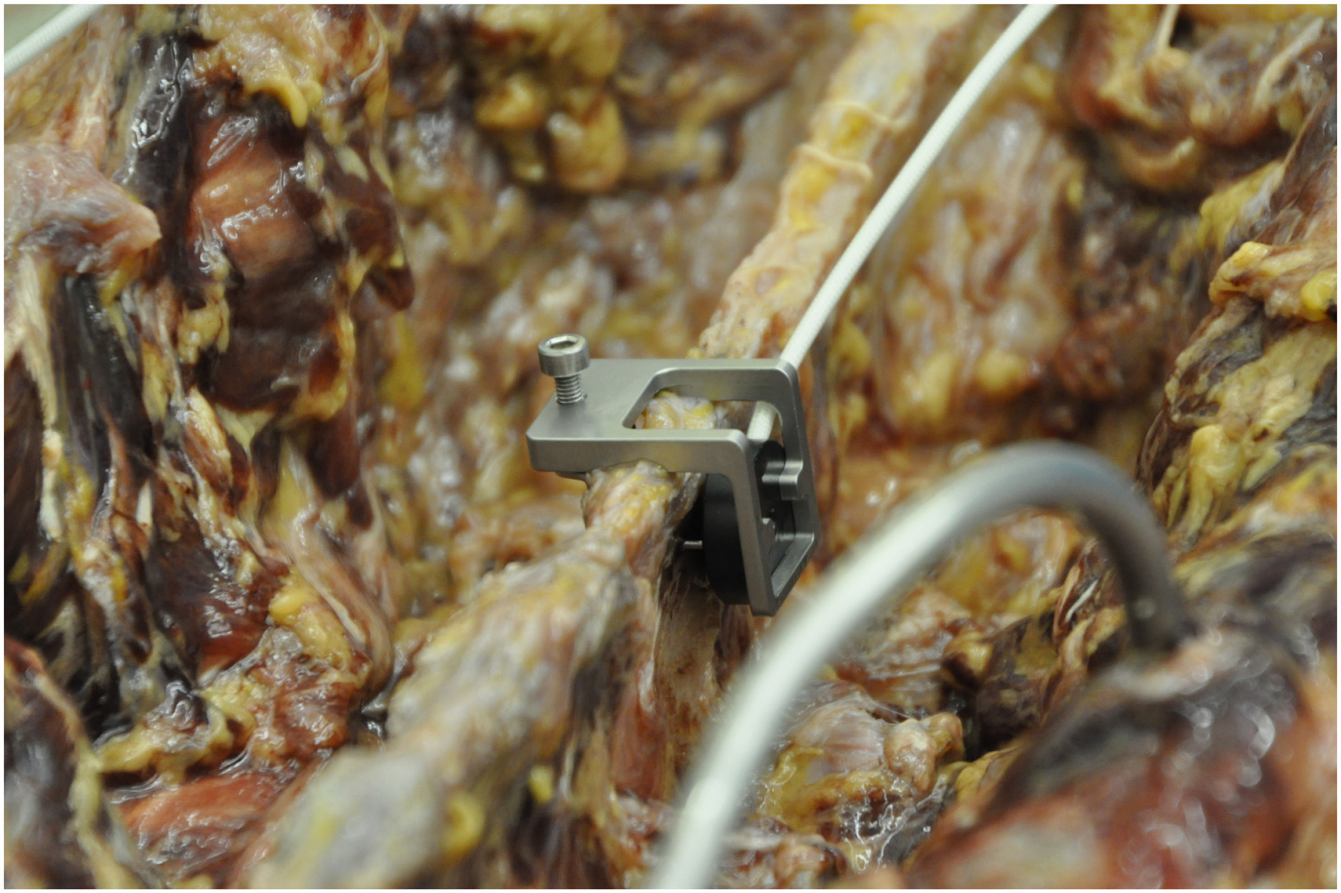
Patient Tracker.

A special field generator was used to generate the electromagnetic field (EMF) (Fig 1). The field generator was placed below the patient (non-sterile area) so that the frame encompassed the entire surgical field. The EM Field in this Study had a spherical size of 670mm. This field generator forms an electromagnetic field in which the instruments in the field fitted with signal coils can be detected. To match the generated electromagnetic field with the image data set and the spine of the cadaver, a reference coil called the patient tracker was attached to the spinous process (Fig 2).

All required and especially developed instruments were free of ferromagnetic substances to prevent measuring and instrument errors. The coils required for navigation are firmly anchored in the instruments using a Luer-Lock system. This makes a standardized sterilization process possible by removing the coil system. Special instruments with hollow cavities had to be developed for this.

The instruments used were a “CenterPointer”, “AwlPointer”, “SpinePointer” and a navigable screwdriver. All instruments were adapted from standard instruments for spine surgery. The CenterPointer is used for surface matching and also to center punch and open the pedicle. The AwlPointer is used to open the vertebral body in the specified direction of trajectory and to determine the screw length required. The SpinePointer makes it possible to palpate the pedicle to detect faulty positions and injuries to the surrounding cortical bone. All instruments are connected with the navigation system by a wire. All pedicle screws used were polyaxial screws with a diameter of 6 mm (S^4^ Spinal System, BBraun, Melsungen, Germany).

### Preoperative Planning

**Fig 3:**
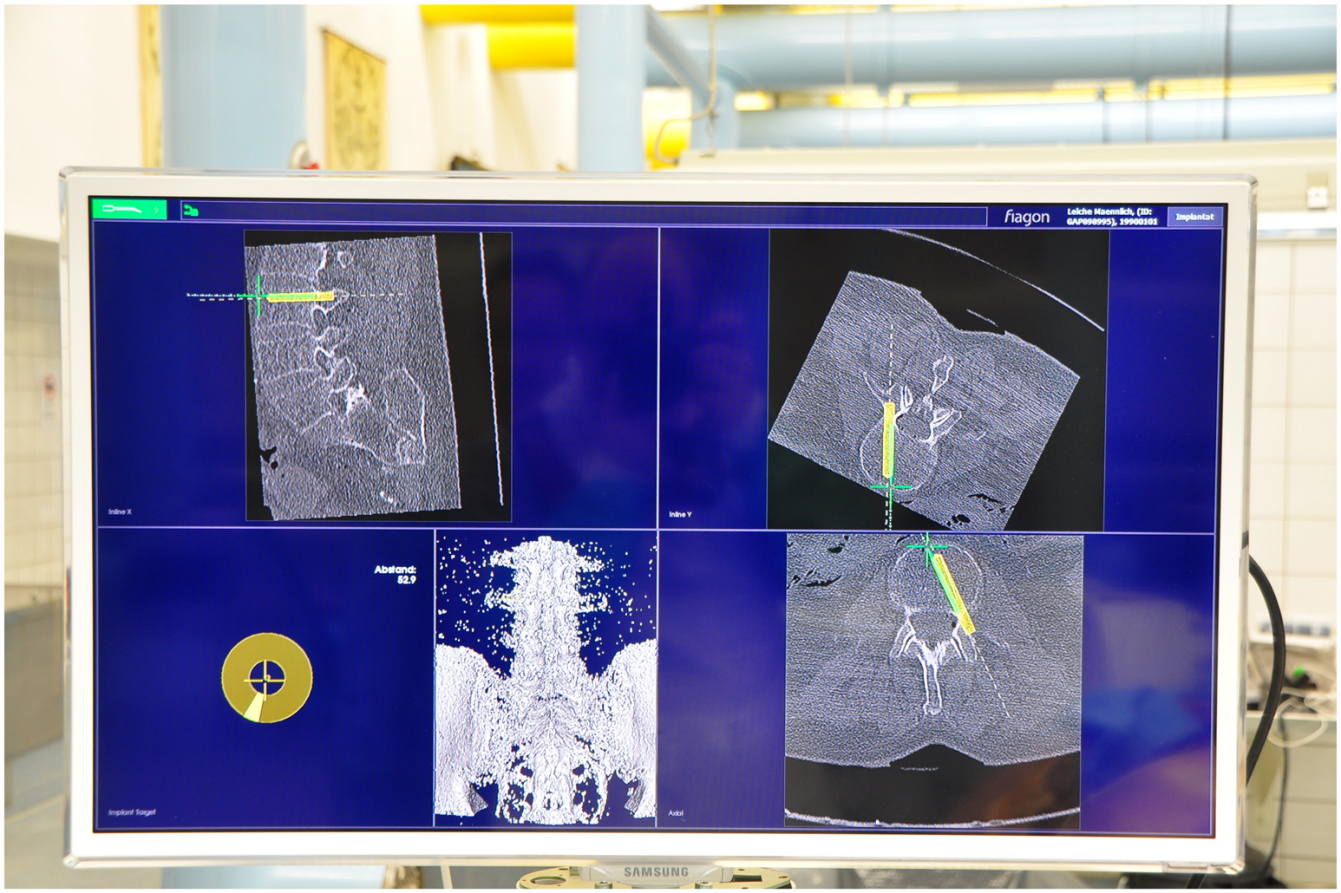
Navigation Planning Tool.

Before the surgical procedure, CT scans with a slice thickness of 1 mm were made of the specimens and a 3D reconstruction was made (Institute for Diagnostic and Interventional Radiology, Düsseldorf University Hospital). These data were used to determine the diameter of the pedicles and the direction of trajectory. The CT data set was stored in DICOM format for further processing and instrumentation in the navigation system. A 3D data set and high-resolution VRT model were calculated by the navigation system (Fig 3).

### Navigation

A total of 20 pedicle screws were inserted in the lumbar spine (bilateral screws at L1–L5) using EMF navigation.

The torsos of the cadavers were placed in prone position on a special, non-metallic, carbon fibre OR table (MAQUET Holding GmbH & Co. KG, Rastatt, Germany) to prevent interference. The field generator was placed below the patient so that the frame encompassed the entire surgical field. Access was via a standard midline incision followed by dissection of the soft tissues up to the screw insertion sites. After preparation of the operation site in the region of the affected segment of the spine, the patient tracker was firmly attached to the spinous process. This step forms the reference in the coordinate space of the navigation unit. The tracker was used to navigate the respective vertebral body.

First, surface matching (10 points) on the respective vertebral body was conducted for the purpose of comparing the CT image data set with the electromagnetic coordinate system. After error calculation of the system and release for navigation, the operator performed an optical check using anatomical landmarks. Navigation was conducted only after this optical check for errors. This was followed by the virtual determination and center punch of the standard insertion point into the pedicle and subsequent opening of the pedicle and the vertebral body using the AwlPointer. Particular attention was paid to positioning the AwlPointer precisely in the center of the pedicle in all planes. A virtual tool was used to calculate the length and diameter of the pedicle screws [Fig 3]. As an additional check, tactile and virtual palpation (optical control on screen) of the pedicle and vertebral body was conducted using the CenterPointer. No differences between tactile and virtual palpation occurred. After this, the screw was inserted under purely navigated guidance. The navigation system enabled real-time visualization of the steps in all spatial planes with various optical options that the operator can adapt as needed.

### Screw Position Evaluation

After the study, the cadavers were again examined by CT scan and evaluated. The results were classified in 5 groups: grade 1 –ideal screw position in the center of the pedicle with no cortical bone injury; grade 2 –acceptable screw position, cortical bone injury with cortical penetration ≤ 2 mm; grade 3: cortical bone injury with cortical penetration 2,1–4 mm, grad 4: cortical bone injury with cortical penetration 4,1–6 mm, grade 5: cortical bone injury with cortical penetration >6 mm [24]. A statistical analysis was made by a team of statistician using an analysis of confidence intervals (SAS 9.3 for MS Windows) because we had no control group.

## Results

A total of 20 pedicle screws and the technical components of the system were evaluated. The evaluation of the screws was performed by 3 evaluators.

The following average pedicle diameters were calculated: L1–6.2 mm, L2–7.4 mm, L3–8.9 mm, L4–11.2 mm, and L5–14.3 mm. The mean pedicle diameter was 8.6 mm. The minimum lumbar pedicle width was 6.1 mm and maximum 12.9 mm; the range was therefore 6.8 mm. The pedicle screws used all had a diameter of 6 mm; all screws were polyaxial screws.

An excellent position (grade 1) was found in 13 pedicles, a minor penetration (grade 2) in 4 pedicles, a moderate deviation (grade 3) in 3 pedicles, and there was no malposition >4,1mm (grade 4 and 5).

Maximum pedicle perforation of no more than 2 mm was thus found in 85% of all screw positions; 17 of 20 screws (85%, 95% confidence interval [62.1%, 96.8%]) were classified as group 1 or 2.

## Discussion and Conclusion

Instrumentation using pedicle screws is a standard procedure in spine surgery [25]. Many different techniques are used for this (free-hand placement, fluoroscopy guidance, 3D fluoroscopy, cone beam CT, intraoperative CT/MRI, etc.) [26–29]. The aim of navigation techniques is to achieve greater accuracy compared with free-hand or fluoroscopy techniques [30–32].

Some disadvantages of the existing methods are that they are prone to errors regarding screw position. In addition, the intraoperative exposure to radiation presents an increased risk to operators, surgery team, and patients due to the intraoperative radiation needed (fluoroscopy-guided insertion technique, 3D fluoroscopy, cone beam CT, etc.) [25, 33]. Another problem is that the operator’s workflow is impaired due to the necessity for optical communication among the components of the system (optical navigational systems) [34].

The disadvantage of navigation systems that work on the basis of detecting optical sensors is that the line of sight can never be interrupted [35]. This limits the operator’s degree of freedom during the procedure, thus also limiting the intuitive use of the operation instruments. The optical navigation technique requires that both, the instruments and the tracker must have passive reflectors or active diodes. The corresponding adaptors that hold the reflectors must be attached to an instrument and also by a clamp in the operation site to be referenced. The disadvantage of this is that the instruments are considerably larger and heavier with poorer ergonomics. In addition, the trackers must be designed so they extend beyond the operation site in order to be detected by the navigation camera. Even very small changes in position due to accidental contact with the reference base can lead to faulty positioning [36].

The aim of this study was the experimental evaluation of a new EMF navigation system for placing pedicle screws. Navigation was carried out using a pre-interventional CT-based data set with an EMF navigation system modified especially for spine surgery.

EMF navigation of pedicle screws using a CT-based data set has not yet been described in literature. The method has several advantages over existing techniques. For one, the intraoperative multiplanar visualization in real time provides a considerable advantage over 2D visualization with a fluoroscopic data set. Furthermore, the system used features easy handling, because nearly unchanged standard instruments can be used for the procedure. It is not necessary to use cannulated screws or K-Wires. In addition, due to the positioning of the patient on the coil generating the EM field and matching using a minimized patient tracker, there are no parts of the navigation system that interfere in the surgical site. The operator’s standard workflow is not impaired. When EMF navigation is used alone, in the current workflow only slight radiation exposure of the operator and surgical team occurs during anatomical positioning of the patient tracker. The slight radiation described here refers to the necessary use of fluoroscopy to determine the correct level, especially for higher regions of the spine. The preoperative CT scan required for navigation can result in an increased radiation dose for the patient compared with other techniques; additional studies are required to compare exposure in different techniques. Another advantage of EMF navigation in comparison with optical methods is the position of the reference coil on the instruments. It is placed near the tip of the instrument, which can result in lower torsion errors.

The evaluation of the pedicle screw position showed a lower rate of faulty positioning (15% grade 3, 0% grade 4 and 5). However, it should be emphasized that due to the large mean size of the lumbar pedicles and a screw size of 6 mm, the allowable error tolerance is greater here than in higher segments of the spine (thoracic and cervical spine).

One limitation of this study is the small sample size and the non-comparative study. Therefore, the results have to be evaluated in future studies with larger sample sizes and the control group.

The relative mobility of the polyaxial screws in the navigated screwdriver must be emphasized. This also results in a slight angle deviation and greater probability of error. The rigidity of the instruments must also be established in larger patient groups. High bending forces can cause problems, especially on the awl, as here the navigation coil is currently not directly attached to the tip of the instrument. This can also cause errors in virtual visualization and navigation.

In summary, the results indicate a good placement technique for pedicle screws using a new EMF navigation system for the lumbar spine. Additional studies with larger populations and smaller pedicle diameters in higher segments of the spine should be conducted. It should be emphasized that the system is easy to handle and does not limit the operator’s workflow or use of standardized instruments. The electromagnetic navigation system has the technical advantage with respect to handling, as no permanent optical connection (line of sight) of the components is required and the operator’s workflow is not impaired. In addition, navigation can take place without intraoperative X-ray guidance, resulting in lower radiation dose for the operator and surgical team. It should be emphasized that special instruments and techniques are employed for EMF navigation that are associated with higher costs than those used in the conventional technique. However, a special operating table (e.g. carbon fiber table) is not required.
